# Experimental and Numerical Investigation of Static and Dynamic Characteristics of Bio-Oils and SAE40 in Fluid Film Journal Bearing

**DOI:** 10.3390/ma15103595

**Published:** 2022-05-18

**Authors:** Muhammad Imran Sadiq, Wan Aizon W. Ghopa, Mohd Zaki Nuawi, Mohammad Rasidi Rasani, Mohd Anas Mohd Sabri

**Affiliations:** Department of Mechanical and Manufacturing Engineering, University Kebangsaan Malaysia, Bangi 43600, Selangor, Malaysia; waizon@ukm.edu.my (W.A.W.G.); mzn@ukm.edu.my (M.Z.N.); rasidi@ukm.edu.my (M.R.R.); anasms@ukm.edu.my (M.A.M.S.)

**Keywords:** journal bearing system, rotordynamics, fluid film bearing, CFD, bio-oils

## Abstract

Mineral-based oils are the market leaders when it comes to their consumption in different types of rotating machines. Recently, a lot of attention has been given to the bio-oils and lubricants due to their better thermophysical, tribological, and environmental characteristics for use in journal bearing and other rotating machines. The superior physical properties of bio-oils have instigated this research in order to evaluate their dynamic characteristics that can cause the harmful dynamic instabilities in rotating machinery. The dynamic characteristics of the fluid film are influenced by temperature, eccentricity ratio, and rotational speed. In this work, the effect of temperature is experimentally measured on the dynamic viscosity of bio-oils and mineral-based oil. The dynamic viscosity measured is then computationally used to estimate the hydrodynamic pressure response of three bio-oils (rapeseed, palm olein, and soybean) and SAE40, a mineral-based oil, to check their performance in the rotor bearing system. It is found that at 40 °C, the hydrodynamic pressure for SAE40 is observed to be 2.53, 2.72, and 3.32 times greater than those of rapeseed, palm olein, and soybean oil, respectively, whereas, at 125 °C, the hydrodynamic pressure for SAE40 is observed to be 8% and 4.3% less than those of rapeseed and palm olein, respectively, but 14% greater than that of soybean oil. Hence, the increasing temperature has less effect on the viscosity and hydrodynamic pressure of bio-oils compared to SAE40. Therefore, for high-temperature applications, the bio-oils can be used with further processing. The superior response of bio-oils is also an indication for better dynamic characteristics.

## 1. Introduction

The governing equation of hydrodynamic lubrication, the Reynolds equation, was established in 1886. From the results of Tower and Petroff, Reynolds derived the basic equation of the hydrodynamic theory of lubrication of a journal bearing from the Navier–Stokes equations using many assumptions. The Reynolds equation is a partial differential equation and cannot be solved in its full form without making certain assumptions to simplify the equation and obtain the solution. There are two general simplifications: the infinitely long bearing (*L/d* = ∞) and the short bearing assumption (∂p/∂z >> ∂p/∂x). In addition, the solution obtained by making the short bearing assumption is much simpler than that from the infinite long bearing assumption [1].

Since then, many works on the solutions of the equation have been published. In journal bearings, the film thickness is not a linear function of the variables such as load, rotational speed, viscosity, stiffness, and damping. For the case of infinitely long bearings, the generalized Reynolds equation, which is a partial differential equation, can be reduced to an ordinary differential equation. The solution of the problem can be immediately derived by integration. However, for journal bearings of finite length, researchers could not succeed in obtaining the general solution for the finite bearing problem. It has even been stated that the exact solution of the Reynolds equation is only possible if the viscosity is constant and the film thickness is a linear function of the variables [2]. The numerical analysis of a plain journal bearing with nanoparticles and lubricating oil added, which uses a modified Reynolds equation considering the time-dependent inertia effects at rotating speeds with turbulence effects, is also investigated. The results show that adding the nanoparticles to an optimized level increases the critical speed, load carrying capacity, and also the overall stiffness and damping of the system [3]. This has become the norm in modern day research to derive and manipulate the Reynolds equation as per the desired operating and boundary conditions. The function of temperature distribution in the bearing oil film is obtained by coupling the Reynolds equation of hydrodynamic lubrication for short bearings of symmetric geometry with the energy equation [4].

It is important to evaluate the performance of journal bearings mathematically by considering different bio-lubricants. This is because the oil whirl and oil whip instabilities are associated mainly with the performance of the journal bearing. For this purpose, the equations are derived for performance evaluation of the journal bearing in terms of load-carrying capacity, oil film thickness, frictional force, and torque. Previously, the evaluation was carried out using the traditional mineral-based lubricants. The development of a mathematical model for the performance evaluation of a journal bearing using bio-lubricants would provide new horizons in terms of bio-lubricants research [5]. In the numerical analysis of journal bearing dynamic characteristics based on CFD, the results show that the film stiffness coefficient increases with the rotational speed, and the speed has little effect on the damping coefficient [6]. It is also important to model the flow in both laminar and turbulent regions to see how the different flow regimes affect the hydrodynamic pressure. It is also reported that revolution speed of the journal has a strong effect on the hydrodynamic film pressure [7].

The most popular mesh-based methods in CFD are the finite difference method (FDM), finite element method (FEM), and finite volume method (FVM) [8]. The most widespread mesh-based approach relies on FVM [9]. The popularity of the FVM in CFD stems from the high flexibility it offers as a discretization method. It owes much of its flexibility and popularity to the fact that discretization is carried out directly in the physical space with no need for any transformation between the physical and the computational coordinate system. The FVM is suitable for complex geometries. The grid defines only the control volume boundaries and need not be related to a coordinate system. Another advantage of the finite-volume method is that mass, momentum, and energy are automatically conserved, as the integral forms of the governing equations are solved. ANSYS Fluent employs the FVM method for the analysis. Many researchers have used the ANSYS Fluent software to carry out the oil film analysis [10,11,12,13,14,15,16]. The temperature variation for different objects and environments can also be measured using CFD [17]. The variation in pressure is also observed when the refinement in grid size is carried out. By changing the grid size, the improvement in accuracy of the results is observed. The average value of pressure is 12% greater than the results obtained by using a coarse grid [18].

The bio-lubricants are environment friendly, renewable, and biodegradable. This gives them an edge over the conventional mineral-based lubricants, which are toxic to the environment and are also non-biodegradable [19,20]. Furthermore, bio-lubricants have good tribological and physical properties, which gives them an edge over the mineral-based lubricants [21]. Therefore, it is important to make use of bio-oils that can contribute to a better environment and enhanced machine life.

The journal bearings can be evaluated by the static and dynamic characteristics under different thermal operating conditions [22]. The static characteristics are evaluated by estimating the oil film pressure, load-carrying capacity, and related forces acting on the oil film, whereas the dynamic characteristics are evaluated by stiffness (*K_xx_*, *K_xy_*, *K_yy_*, *K_yx_*) and damping coefficients (*C_xx_*, *C_xy_*, *C_yy_*, *C_yx_*). The cavitation and journal whirl are used to evaluate the static characteristics, dynamic characteristics, and stability of journal bearings [23]. The majority of the testing carried out to evaluate the performance of bio-oils is performed through the use of a high-frequency reciprocating test rig [20]. Therefore, it is important to develop a test rig to experimentally test these oils to obtain more accurate and precise results. Bio-oils are reported to have good stiffness characteristics when tested against the synthetic and mineral-based oils [24]. Similarly, in gas-lubricated micro-bearings, the temperature increase has an inverse effect on the viscosity of gas, which ultimately weakens the load-carrying capacity and stability of micro-bearings [22].

Thus, in this paper, the performance of bio-oils (rapeseed, palm olein, and soybean) and SAE40 is computationally evaluated in terms of hydrodynamic pressure, in order to determine how the bio-oils behave under different dynamic conditions in fluid film journal bearings. This is novel area of research that has not yet been experimentally and numerically simulated for bio-oils and lubricants. The short bearing assumption is used for analysis purposes. Furthermore, using different eccentricity ratios, the dynamic characteristics are also evaluated.

## 2. Theoretical Background

### 2.1. Static Performance Evaluation

The nonlinear fluid film forces generated by the journal bearing may be derived by considering the solution to the Reynolds equation. The Reynolds equation for laminar, iso-viscous, and iso-thermal flow may be written as [5]:

(1) $$\frac{\partial }{\partial x}\left(\frac{h^{3}}{\eta }\;\frac{\partial p}{\partial x}\right)+\frac{\partial }{\partial z}\left(\frac{h^{3}}{\eta }\;\frac{\partial p}{\partial z}\right)=6\;U\;\frac{\partial h}{\partial x}+12\;\frac{\partial h}{\partial t}$$

where *h* is the film thickness, *p* is the pressure developed in the film, and *η* is the oil film viscosity. Similarly, another important parameter to define a bearing that combines speed and load effects is often termed as the bearing characteristic number or the Sommerfeld number *S*:

(2) $$S=\frac{\;N\;D\;L}{W}\;{\left[\frac{R}{C}\right]}^{2}$$

where *η* is the viscosity of the lubricant, *N* is the speed of shaft rotation, *D* is the diameter of the bearing, *L* is the bearing length, *W* is the applied load, *R* is the shaft radius, and *C* is the radial clearance.

In addition, the eccentricity ratio *ε*, is given by:

(3) $$\varepsilon =\frac{e}{C}$$

where *e* is the eccentricity and *C* is the radial clearance. The eccentricity ratio has a great impact on the hydrodynamic pressure, load carrying capacity, stiffness, and damping coefficients. The effect of eccentricity is also discussed in detail on the abovementioned parameters.

### 2.2. Dynamic Performance Evaluation

Assuming small changes in displacement and velocity and steady-state equilibrium position, the dynamics of the oil film is described by linearized stiffness and damping coefficients. The total forces in the *x* and *y* direction become [25]:

(4) $$\left\{\begin{array}{c} F_{x}\\ F_{y} \end{array}\right\}=-\left[\begin{array}{cc} K_{xx} & K_{xy}\\ K_{yx} & K_{yy} \end{array}\right]\left\{\begin{array}{c} ∆x\\ ∆y \end{array}\right\}-\left[\begin{array}{cc} C_{xx} & C_{xy}\\ C_{yx} & C_{yy} \end{array}\right]\left\{\begin{array}{c} ∆\dot{x}\\ ∆\dot{y} \end{array}\right\}$$

(5) $$F_{x}=-(K_{xx}∆x+K_{xy}∆y+C_{xx}∆\dot{x}+C_{xy}∆\dot{y})$$

(6) $$F_{y}=-(K_{yx}∆x+K_{yy}∆y+C_{yx}∆\dot{x}+C_{yy}∆\dot{y})$$

where *K_ij_* and *C_ij_* are the stiffness and damping coefficients, respectively. The negative sign in the equation shows that the force is acting on the journal. The eight stiffness and damping coefficients depend on the steady-state condition of the journal and, in particular, the rotational speed.

From the above equations, we have two direct or principal stiffness coefficients:

(7) $$K_{xx}=∆F_{x}/∆x$$

(8) $$K_{yy}=∆F_{y}/∆y$$

where each stiffness coefficient relates the change in force in one direction due to the change in displacement in the same direction.

Similarly, two direct or principal damping coefficients are also present:

(9) $$C_{xx}=∆F_{x}/∆\dot{x}$$

(10) $$C_{yy}=∆F_{y}/∆\dot{y}$$

where the damping coefficients relate the small change in force due to a small change in velocity. In addition, the cross-coupled damping terms are equal:

(11) $$C_{xy}=C_{yx}$$

The cross-coupled stiffness coefficients can be defined as:

(12) $$K_{xy}=∆F_{x}/∆y$$

(13) $$K_{yx}=∆F_{y}/∆x$$

The coefficient *K_yx_* relates to a vertical force due to a displacement in the *x* direction. Thus, the horizontal and vertical directions are coupled. Almost all structures have cross-coupled stiffness terms, but most are symmetric in nature, meaning *K_xy_* = *K_yx_*. The cross-coupled stiffnesses are detrimental because, instead of damping the whirling motion of the rotor such as the direct damping, these cross-coupled stiffnesses combine to produce a force in the whirl direction, increasing the shaft vibration. When the direct damping is unable to dissipate the energy induced by the cross-coupled stiffness force, the natural frequency (generally, the first natural frequency with forward whirling direction) will become unstable, causing the journal to whirl at this frequency [25].

In this work, the dimensionless stiffness and damping coefficients are determined for various eccentricity ratios. These coefficients are important in defining the overall dynamic characteristics of the fluid film bearing under different loading conditions.

## 3. Materials and Methods

### 3.1. Geometrical Model

**Figure 1:**
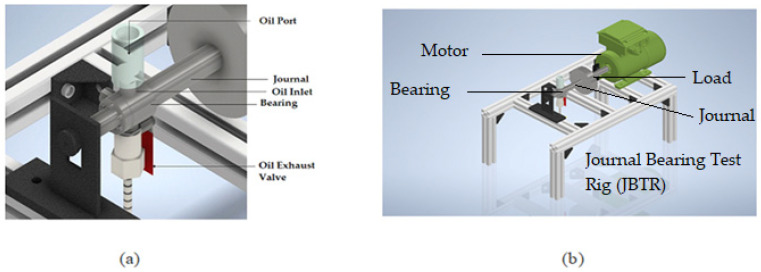
(**a**) Journal bearing schematic; (**b**) overall diagram of Journal Bearing Test Rig (JBTR).

In this work, the journal bearing is modeled by taking the following design values. The short bearing assumption is used where *L/d* ≤ 0.5. In addition, the load applied on the shaft is in the form of a disk, as shown in Figure 1b. The specifications of the journal bearing test rig (JBTR) are given in Table 1.

Figure 1 shows a schematic diagram of the Journal Bearing Test Rig (JBTR). Figure 1a shows the highlighted area where the oil is supplied to the bearing and the oil film is formed, whereas Figure 1b shows the overall JBTR.

**Figure 2:**
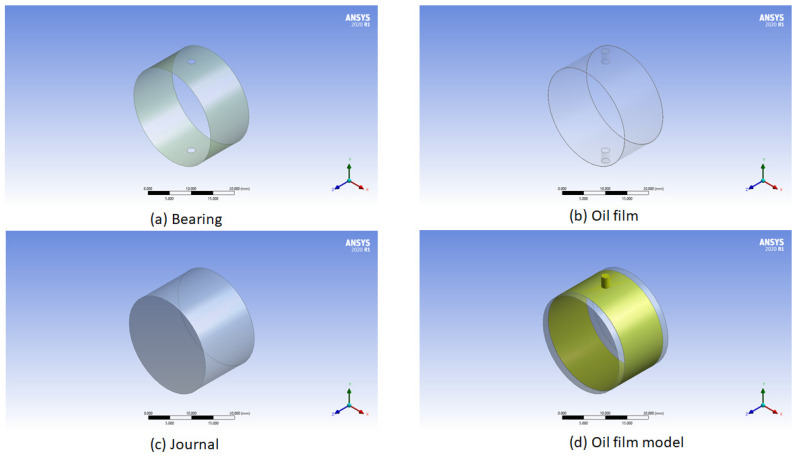
Ansys oil film model.

Figure 2 shows the simplified Ansys model for the fluid film model. Figure 2a represents the bearing, Figure 2b represents the oil film, Figure 2c represents the journal, and Figure 2d represents the overall model. In this way, the oil film model is developed. In the further discussion, only the oil film model is discussed for hydrodynamic pressure variation with temperature and viscosity.

### 3.2. Dynamic Viscosity Measurement

**Figure 3:**
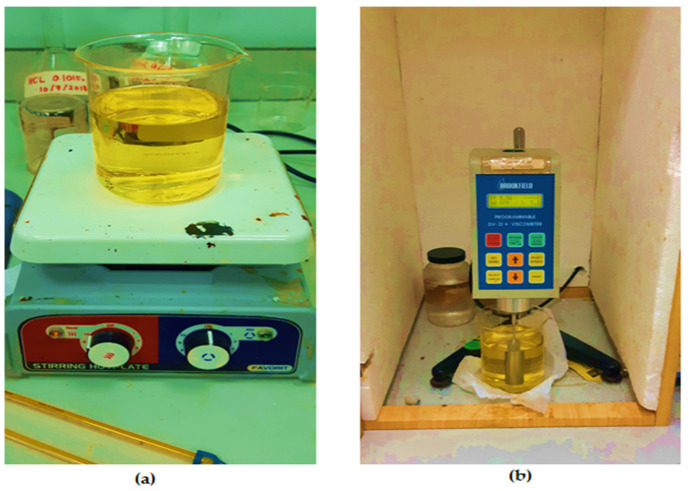
(**a**) Hot plate; (**b**) Viscometer for viscosity measurement.

Dynamic viscosity is the measure of the shear stress per unit area required before an oil sample begins to deform. Lubricants are characterized by their viscosity as a function of temperature, pressure, and shear rates. However, viscosity is largely influenced by the working temperature of the liquid [26]. The increase in temperature decreases the viscosity of the fluid. The viscosity of a liquid is measured using a device called a viscometer. In order to test the oil viscosity of the oil samples, the Brookfield Viscometer is used, as shown in Figure 3. The hot plate is used to heat the oil to the desired temperature, whereas the rotational viscometer is then used to measure the dynamic viscosity at the given temperature. ASTM standard D4402–02 is employed to carry out the procedure.

Table 2 shows some important properties of three bio-oils and SAE40 oil. Three bio-oils have almost similar flash points, which is almost 90 °C higher compared to that of SAE40. This is also an important factor when we consider that the operational range of machines is at higher temperatures, such as 120~200 °C [27]. Similarly, the density of bio-oils is also greater than that of SAE40 oil.

### 3.3. Computational Method

The Navier–Stokes equation, mass and momentum equation, and energy conservation equations are solved in steady-state, taking gravity forces into account. The model is established using ANSYS Fluent. The operating pressure is taken as an absolute 101,325 Pa at the inlet and 0 Pa at the outlet. In this work, the results are obtained by assuming a laminar flow. For the hydrodynamic pressure measurement for bio-oils and SAE40, the viscosity is not kept constant. Instead, the variable viscosity values are used, measured at different temperatures for different types of oils. The bearing shell is modeled as a stationary wall. The journal is modeled as a moving wall with a rotational speed of 3000 rpm. The rotational axis origin is set to the value of eccentricity. The lubricant inlet is modeled as pressure inlets and the lubricant outlet is modeled as pressure outlets. For greater accuracy, a convergence criterion of the order of 10^−6^ is used for all residual terms. The limitations for the Ansys fluent solver are that it uses constant viscosity unless the values are changed by the user.

### 3.4. Mesh Refinement

Mesh refinement is carried out in order to obtain the accurate results of various parameters under consideration in ANSYS Fluent. At larger mesh size, the results are generally not accurate. As the mesh size decreases, the results tend to become more accurate, although the computational time also increases.

**Figure 4:**
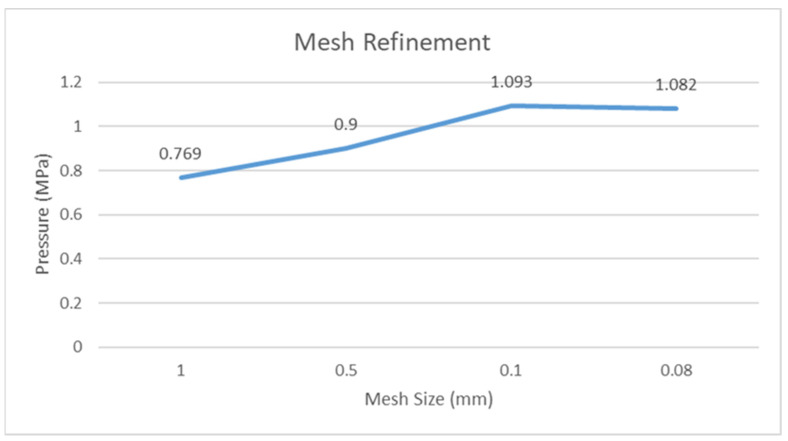
Effect of mesh size on the maximum hydrodynamic pressure (ANSYS Fluent).

Figure 4 shows the pressure variation for different mesh sizes measured in ANSYS Fluent. As the mesh size becomes finer, the pressure value increases. At the same time after reaching the optimum value, the pressure does not increase any further. Thus, in this case, 0.1 mm is the value at which accurate results can be achieved. Further decreasing the mesh size from here on will only increase the burden on the computational system. As mentioned by [18], the increase in pressure is observed when the mesh refinement is carried out. An increase of approximately 30% in pressure is observed from a coarser mesh to a finer mesh.

Therefore, based on the dynamic viscosity values at different temperatures, the CFD technique is used for further evaluation of bio-oils and SAE40 for the hydrodynamic pressure distribution.

## 4. Results and Discussion

### 4.1. Effect of Temperature on Dynamic Viscosity

Increasing temperature has an effect on the dynamic viscosity of oils. As the temperature increases, the viscosity decreases. This is further elaborated in Figure 4 below.

**Figure 5:**
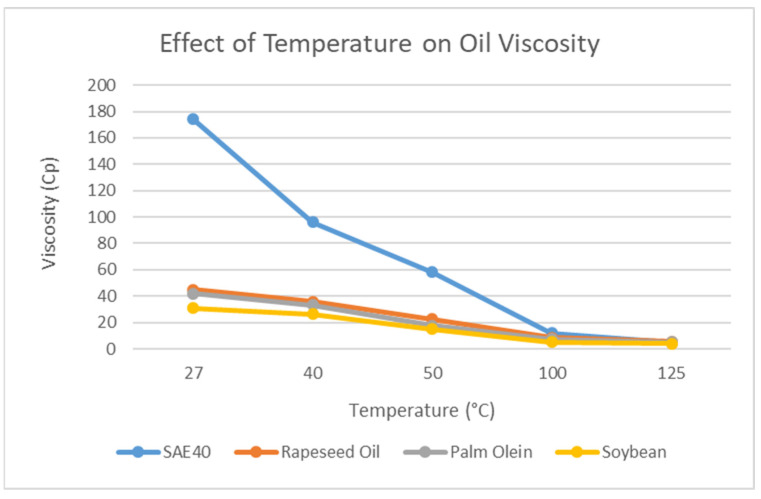
Effect of temperature on oil viscosity.

The dynamic viscosity of the three types of bio-oils and SAE40 is measured from 27 °C to 25 °C. The experimental measurement of dynamic viscosity is performed using the viscometer. From Figure 5, we can see the effect of temperature on the dynamic viscosity of different types of oils. There is sharp decrease in viscosity observed for SAE40 from room temperature to 50 °C and from 50 °C to 100 °C, as reported by [29]. The behavior of three bio-oils is observed as quite similar with increasing temperature as the viscosity drop is much less compared to SAE40. In addition, the value of viscosity for SAE40 (4.8 cP) drops below those of rapeseed oil (5.3 cP) and palm olein (5 cP) at 125 °C. This is an important observation that leads to a drop in hydrodynamic pressure and load-carrying capacity of oil films at higher temperatures for SAE40.

### 4.2. Effect of Eccentricity Ratio ε on Stiffness K and Damping C Coefficients

**Figure 6:**
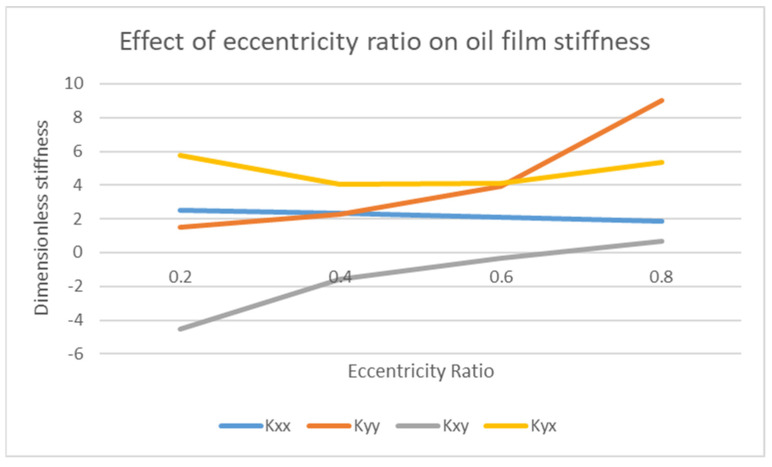
Effect of eccentricity ratio *ε* on dimensionless stiffness *K*.

The eccentricity ratio also affects the stiffness and damping coefficients. The stiffness and damping coefficients play an important role in defining the dynamic response of the system. This is further explained in Figure 5 and Figure 6.

In Figure 6, the effect of eccentricity ratio against the dimensionless stiffness is plotted. All the four stiffnesses have different trends as the stiffnesses tend to vary for each dimension and condition. The cross-coupled stiffness terms *K_xy_* and *K_yx_* are the prime cause of rotordynamic instability. Therefore, it is very important to keep these terms as low as possible. The stiffness values are plotted for different eccentricity ratios. Depending on the condition of eccentricity ratio (load, rotational speed, length, and diameter), the concerned value of *K_ij_* can be used. The dimensionless stiffness coefficient *K_ij_* can be converted to dimensional stiffness coefficient *k_ij_* by the given formula:

(14) $$K_{ij}=\frac{k_{ij}\;C}{W}$$

where *C* is the radial clearance and *W* is the load on the bearing.

**Figure 7:**
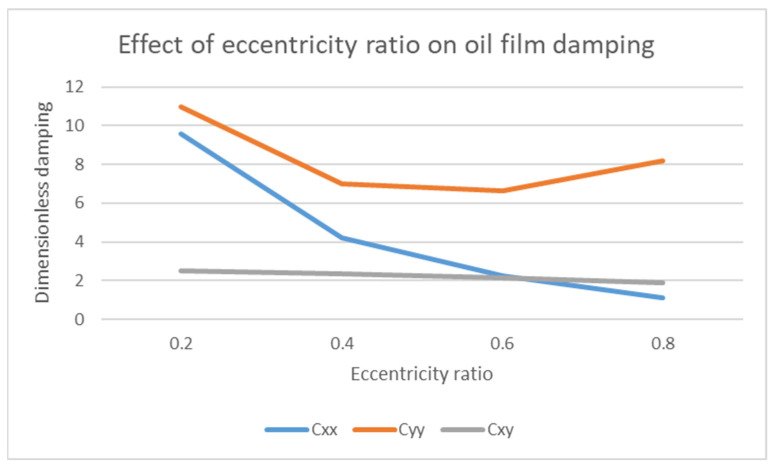
Effect of eccentricity ratio *ε* on dimensionless damping *C*.

In Figure 7, the effect of eccentricity ratio against the dimensionless damping is plotted. For damping coefficients, *C_xy_* and *C_yx_* have the same value. The damping values are plotted for different eccentricity ratios. Depending on the condition of eccentricity ratio (load, rotational speed, length, and diameter), the relevant value of *C_ij_* can be used. The dimensionless damping coefficient *C_ij_* can be converted to dimensional damping coefficient *c_ij_* by the given formula:

(15) $$C_{ij}=\frac{c_{ij}\;C\;\Omega }{W}$$

where Ω is the rotational speed. The use of these eight stiffness and damping coefficient data values makes possible the determination of journal bearing characteristics on the stability of the rotor bearing system. The cross-coupled terms are the actual source of the dynamic instability effect in the rotating system. Therefore, these terms must be kept in the range of (0.4–0.6) for eccentricity ratio in order to obtain dynamic coefficients in the intermediate range, which is deemed as the safe range [30]. Another main point related to eccentricity ratio is that, if it is increased so that it approaches 1, the probability of self-excited vibrations (oil whirl and whip) also increases, as reported by [31].

### 4.3. Numerical Analysis

Hydrodynamic pressure is measured for bio-oils and SAE40 under different operating conditions of temperature and viscosity using ANSY Fluent. This is further explained in the figures below.

**Figure 8:**
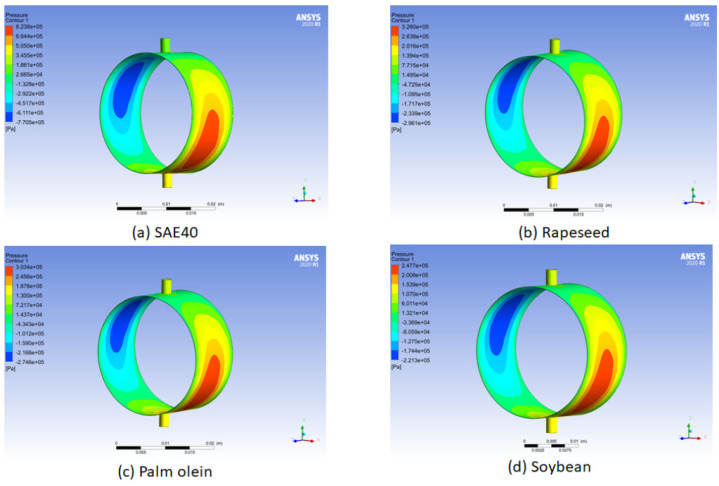
Hydrodynamic pressure distribution.

**Figure 9:**
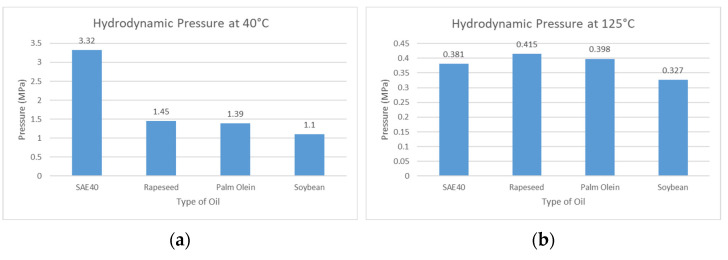
Comparison of maximum hydrodynamic pressure.

From Figure 8, the hydrodynamic pressure distribution for bio-oils and SAE40 from the ANSYS Fluent post processing can be seen. Figure 8a gives the pressure distribution for SAE40. Similarly, Figure 8b–d show the pressure distribution of three bio-oils (rapeseed, palm olein, and soybean), respectively. For the pressure distribution on the bearing, the convergent oil wedge and divergent oil wedge can be seen in red and blue zones, respectively. It shows that the hydrodynamic pressure is higher for SAE40 at 40 °C compared to three bio-oils. This is further explained in Figure 9.

In Figure 9a,b, the maximum hydrodynamic pressure is compared between the bio-oils and SAE40. Figure 9a shows data at 40 °C whereas, Figure 9b shows data at 125 °C. The highest value of maximum hydrodynamic pressure is noted for SAE40 because of the high value of viscosity at a temperature of 40 °C. However, this value of maximum hydrodynamic pressure decreases for SAE40 at 125 °C. Hence, at a temperature of 125 °C, the load-carrying capacity also decreases due to the low viscosity for SAE40, as reported by [22].

**Figure 10:**
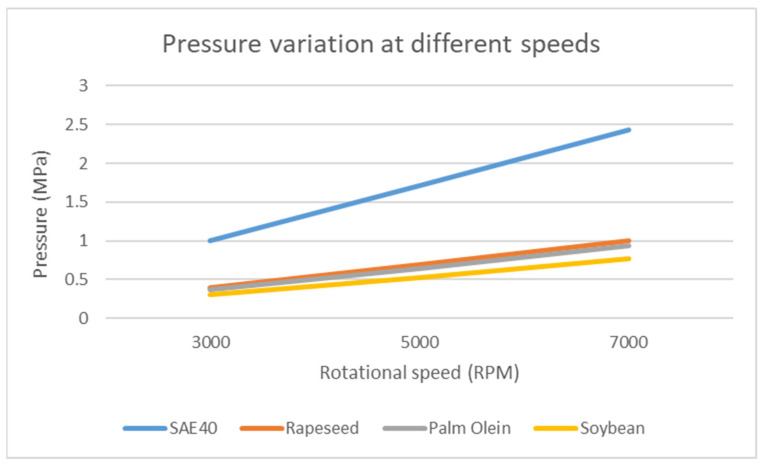
Effect of RPM on hydrodynamic pressure (ANSYS Fluent).

Figure 10 gives the general trend of increasing speed of the journal on the hydrodynamic pressure using ANSYS Fluent. At low speed, due to the lubricant presence with suitable thickness, the bearing surface does not bear any pressure. As the speed of the journal increases, the hydrodynamic pressure also tends to increase for constant viscosity, and the bearing bears more pressure. This is also reported by [31,32]. This trend is observed when the viscosity is constant with increasing rotational speed of the journal, whereas, under actual operating conditions, this does not stand true. As the rotational speed of the journal increases, the viscosity also tends to decrease due to the increasing temperature of the lubricant. Thus, the trend shown in Figure 5 will be observed.

**Figure 11:**
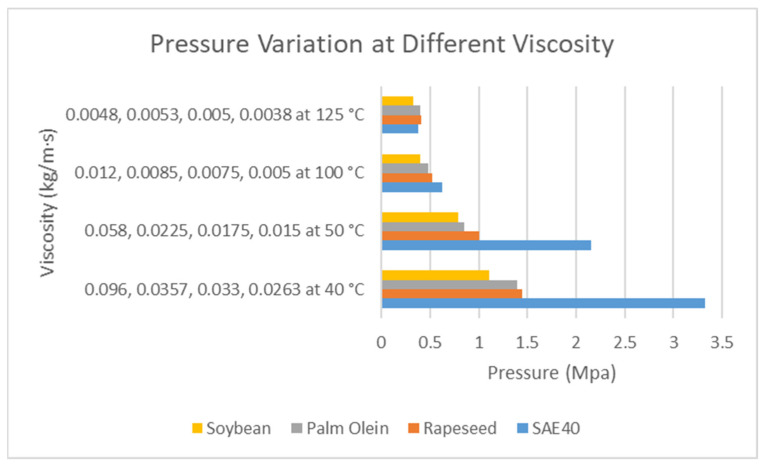
Pressure variation with variable viscosity.

In Figure 11, the effect of viscosity can be seen on the hydrodynamic pressure. It is important to mention here that with the increasing temperature, the viscosity decreases. The four values at each temperature correspond to SAE40, rapeseed, palm olein, and soybean. From Figure 4, we obtain the response of viscosity variation as the temperature increases. The viscosity of SAE40 decreases sharply compared to the three bio-oils. It can be seen here that the effect of viscosity on pressure is quite similar to that in Figure 5. The rise in temperature decreases the dynamic viscosity, which ultimately decreases the hydrodynamic pressure and the load-carrying capacity of the oil film, as reported by [22]. At lower viscosity, the adjacent layers of the oil film are not too tightly bonded together, and they tend to shear more at lower viscosity as compared to the higher value of viscosity. Hence, the load-carrying capacity of the oil film also sees a decline at higher temperature as the loading-carrying capacity is also dependent on viscosity. From the CFD analysis, it is also observed that at higher temperatures, the hydrodynamic pressure response of bio-oils is better than that of SAE40. In addition, in order for bio-oils to be utilized for industrial applications for better physical and dynamic characteristics, additives need to be added in order to comply with the standards [33,34].

Overall, from the CFD results, rapeseed oil and palm olein have shown superior responses in comparison with SAE40, showing a lower decrease in viscosity and giving higher values of hydrodynamic pressure at higher temperatures. These oils can be specifically considered for applications where the steady-state temperature of the journal bearing oil film is above 100 °C.

## 5. Conclusions

Based on the CFD study to measure the hydrodynamic pressure response, bio-oils have shown a superior response. Similarly, from the results, it can be concluded that:

◦ The dynamic viscosity drop is much less for bio-oils compared to SAE40. The stable behavior of bio-oils against the increasing temperature makes them suitable for high-temperature applications in journal bearings.
◦ At 40 °C, the hydrodynamic pressure for SAE40 is observed to be 2.29, 2.39, and 3.01 times greater than those of rapeseed, palm olein, and soybean oil, respectively. Hence, the load-carrying capacity of oil film for SAE40 is also higher as the viscosity is higher compared to bio-oils.
◦ By contrast, at 125 °C, the hydrodynamic pressure for SAE40 is observed to be 8% and 4.3% less than those of rapeseed and palm olein, respectively, but 14% greater than that of soybean oil. This decrease in hydrodynamic pressure causes the decline in load-carrying capacity of oil film for SAE40 compared to bio-oils.
◦ Stiffness and damping coefficients are also investigated for different eccentricity ratios. Depending on the operating parameters (load, rotational speed, and viscosity), the value of eccentricity ratio can be calculated. The eccentricity ratio is important in evaluating the dynamic characteristics (stiffness and damping).

This shows how the temperature rise affects the performance of mineral-based lubricants more compared to bio-oils. Similarly, changing the eccentricity ratio also changes the stiffness and damping coefficients, which can have an effect on the dynamic stability of the system. Furthermore, the bio-lubricants used here are unprocessed compared to the mineral-based lubricant SAE40. The treatment of bio-oils according to operating conditions and requirements can also enhance their physical and dynamic properties.

## Figures and Tables

**Table 1:** Specifications of the Journal Bearing Test Rig (JBTR).

No	Description	Specification
1	*L*, bearing length	12.5 mm
2	*D*, inner diameter for plain bearing	25.14 mm
3	*d*, shaft diameter	25 mm
4	Weight of journal	9 N
5	*W*, Weight of load	25 N
6	*C_T_*, total clearance	0.14 mm
7	*C*, radial clearance	0.07 mm
8	*ε*, Eccentricity ratio	0.2–0.8
9	*L/d* ratio (short bearing assumption)	0.5
10	Operating speed	1000~3000 rpm

**Table 2:** Physical properties of oil samples [28].

Properties	SAE40	Palm Olein	Rapeseed	Soya Bean
Flash point (°C)	235	324	326	330
Specific heat, Cp (kJ/kg·C)	2.53	1.9	1.96	1.88
Thermal conductivity (W/m·C)	0.145	0.172	0.168	0.185
Density at 15 °C (g/cm^3^)	0.890	0.912	0.915	0.924

## Data Availability

Data are available upon request from the corresponding author. These data are not commercially available, due to privacy issues.

## Nomenclature

*h*	oil film thickness
*C*	radial clearance
*N*	rotational speed
*D*	diameter of bearing
*L*	length of bearing
P	pressure
*U*	velocity of fluid
*e*	eccentricity
*ϕ*	attitude angle
*ε*	eccentricity ratio
*K_xx_, K_yy_*	dimensionless direct stiffness
*K_xy_, K_yx_*	dimensionless cross-coupled stiffness
*C_xx_, C_yy_*	dimensionless direct stiffness
*C_xy_, C_yx_*	dimensionless cross-coupled stiffness
